# Housekeeping Tip: Long-Term Frequent Use of Some Household Products May Affect Heart Rate Variability

**DOI:** 10.1289/ehp.120-a284a

**Published:** 2012-07-02

**Authors:** Julia R. Barrett

**Affiliations:** Julia R. Barrett, MS, ELS, a Madison, WI–based science writer and editor, has written for *EHP* since 1996. She is a member of the National Association of Science Writers and the Board of Editors in the Life Sciences.

Use of household cleaning products may be accompanied by inhalation of volatile organic compounds (VOCs) and the secondary pollutants created by their reactions with other airborne compounds. Indoor exposure to these chemicals has been associated with conditions ranging from mucosal membrane irritation to poor concentration. A new study suggests that heavy use of some household cleaning and air-freshening products may have an adverse effect on cardiovascular function [*EHP* 120(7):958–964; Mehta et al.].

The study drew on data collected through the Swiss Cohort Study on Air Pollution and Lung and Heart Diseases in Adults (SAPALDIA), a prospective cohort study involving 9,561 adults that began in 1991. Upon enrollment, participants underwent medical examinations and completed a detailed health questionnaire. A second round of assessments occurred in 2001–2003, for which one subset of 3,255 participants completed a detailed questionnaire about household cleaning activities, and another subset of 1,846 participants aged 50 years or older underwent 24-hour electrocardiogram (ECG) monitoring. The current study was a cross-sectional analysis of 581 participants aged 50 years or older, mostly women, who completed both the questionnaire and the ECG monitoring. Many of the participants were full-time homemakers.

The ECG monitoring focused on four summary measures of heart rate variability (HRV), reduced HRV being a marker of cardiac dysfunction. Participants reported use of several different household sprays and scented products. Lung-function test results and self-reported symptoms reflected the state of participants’ pulmonary health. Statistical analysis assessed HRV measures in association with frequency of use of cleaning sprays, air-freshening sprays, and scented products, as well as with the number of different sprays used weekly. The investigators adjusted for individual health and cardiovascular risk factors, as well as for alternative sources of other indoor air pollutants.

Long-term frequent use of all product types was associated with reduced HRV, with the strongest association for air-freshening sprays. Associations were stronger among individuals with evidence of obstructive lung disease (not including asthma). Limitations include the cross-sectional design (which represents only a snapshot in time), reliance on self-reported exposures, potential selection bias, and uncontrolled confounding. However, the reported associations were strong, suggesting widespread heavy use of these types of products could have a significant public health impact if the observed associations represent casual effects. More rigorous study is needed, including longitudinal observations, direct exposure assessment, and chemical characterization of VOCs and secondary pollutants in homes’ indoor and outdoor air.

